# Comparison of clinical outcome of laparoscopic versus open appendectomy, single center experience

**DOI:** 10.1016/j.heliyon.2018.e00635

**Published:** 2018-05-24

**Authors:** Mitsugi Shimoda, Tsunehiko Maruyama, Kiyotaka Nishida, Kazuomi Suzuki, Tomoya Tago, Jiro Shimazaki, Shuji Suzuki

**Affiliations:** Department of Gastroenterological Surgery, Tokyo Medical University, Ibaraki Medical Center, Japan

**Keywords:** Surgery

## Abstract

**Introduction:**

Laparoscopic appendectomy (LA) is now a treatment of choice in patients with appendicitis. This study compares the treatment outcomes of LA and open appendectomies (OA) in our department.

**Patients and Methods:**

From January 2006 to April 2016 a total of 185 patients underwent appendectomy at our institution. We divided the patients into two groups; LA group (LAG) and OA group (OAG). Following parameters were analyzed: age, gender, preoperative clinicolaboratory characteristics, operative factors, interval appendectomy, length of hospital stay (LHS), and surgical site infections (SSI).

**Results:**

There were 93 patients in LA G and 92 in OAG. According to the Univariate analysis, there were statistically significant differences among age (p = 0.037), LHS (p = 0.0001), duration till resuming oral intake (p = 0.016), blood loss (p = 0.038), SSI ratio (p = 0.044) and CRP level (p = 0.038) between the LAG and the OAG. According to the Multivariate analysis, blood loss (p = 0.038) and LHS (p = 0.023) were significantly different between both groups.

**Conclusion:**

LA was decreasing blood loss and LHS.

## 1 Introduction

Since its first description in the early 1990s laparoscopic appendectomy (LA) has advanced to becoming the treatment of choice for acute and chronic appendicitis, with increasing numbers of procedures performed last decade [1]. Several reports have described the superiority of LA compared to open appendectomy (OA) [2, 3, 4, 5, 6]. Advantages LA has compared to OA are reduced postoperative pain, better cosmetic appearance post-surgery, and increased chance of early discharge. However, whether LA or OA is the best procedure for patients suffering from acute appendicitis still remains unclear.

In this retrospective study, we compared the treatment outcomes and perioperative clinical factors of LA and OA performed in our department to determine the best procedure for appendectomy.

## 2 Materials & methods

### 2.1 Patient population and selection

We retrospectively analyzed 185 Japanese patients who were diagnosed with appendicitis and underwent LA or OA, and their operational outcomes, from January 2010 to January 2016. In total 185 patients with appendicitis defined as a phlegmonous, gangrenous transformation of the appendix, or perforation of the appendiceal wall (macroscopic or microscopically proven by histological report), were included in this retrospective study.

Prior to surgery all patients were subjected to clinical examination and blood and urine screening. In all of patients, plain computed tomography (CT) scan was conducted to ensure correct diagnosis. Same patients received non-surgical treatment before surgery because most of the patients did not have remarkable abdominal pain at the time of outpatient clinic. After the onset of acute appendicitis, a case requiring more than 7 days until surgery was defined as interval appendectomy.

Prior to surgery each patient was given intravenous single shot antibiotic treatment consisting of a first-generation cephalosporin, and in cases of perforation, gangrene, and/or abscess formation, second-generation cephalosporin was added.

### 2.2 LA and OA procedure

All operations were performed by a team of two surgeons who were experienced in the open as well as the laparoscopic approach. Three ports were inserted in LA G, one 10-mm port (camera port) in umbilicus at first and two 5-mm ports in right lower quadrant and suprapubic portion, respectively. The mesoappendix was cut with SonoSurg (Olympus Corporation, Japan) and the appendiceal stump was closed with endoloop (Ethicon, USA). If patients need to convert from LA to OA, we prepared a laparotomy at right paratectal incision. In OA, all approaches were performed at the right paratectal incision. In the case of perforation or ascites, we inserted a drain at the stump of appendectomy or utilized a Douglas pouch.

### 2.3 Study design

We evaluated clinicopathologic and operative factors in all of the 185 patients. We divided into two groups LA group (LAG) and OA group (OAG), and clinicopathologic factors were selected and compared between two groups. These included age, gender, albumin, C-reactive protein (CRP), neutrophil to lymphocyte ratio (NLR), white cell counts (WBC), operative factors (time and amount of blood loss), length of hospital stay (LHS), day from diagnosis to operation (DDO) and 30-day morbidity focusing on occurrence of surgical site infections (SSI). The NLR was defined as the value calculated by dividing the absolute neutrophil count by the absolute lymphocyte count [7].

The patients were then compared regarding the surgery duration, postsurgical symptoms such as pain, wound infection, DDO, LHS and SOI. SSI was graded using the Clavien–Dindo classification of surgical complications [8].

The study was approved by the research and ethics committee at the Tokyo Medical University, Ibaraki Medical Center (Number: 16–34). The patients who completed follow-up were included in the study.

### 2.4 Statistics

Statistical analyses were performed with the SPSS statistical software package (version 13.0; SPSS Inc., Chicago, IL). Median was used to define laboratory parameters such as, age, CRP, Alb, WBC, NLR, amount of bleeding operating time and LHS. Univariate and multivariate analyses were performed to clarify the laboratory parameter and clinical factors most significantly associated with LA and OA. Univariate analyses, Mann-Whitney U-test, and Fisher's exact test were utilized, and Odds ratios with 95% CI were calculated using logistic regression model analyses. P values of less than 0.05 were considered to be statistically significant.

## 3 Results

From January 2010 to April 2014, 92 patients underwent LA and 93 patients OA for appendicitis. Nine of 93 patients (9.7%) experienced conversion from LA to OA.

Age was significantly lower in the LA G than in OA G (P = 0.037). Among the preoperative blood chemistry data, only CRP level was significantly lower in the LA G than in OA G (P = 0.024: Table 1). Operating time did not differ between both groups, however, blood loss was significantly lower in LA G than in OA G (P = 0.038). In LA G, LHS and SOI were significantly shorter than in OA G (P < 0.001 and 0.016), and SSI ratio was significantly lower than in OA G (P = 0.044). In OA G, gangrenous appendicitis ratio was significantly higher than in LA G (P = 0.003) (Table 2).

**Table 1:** The characteristics before surgery according to the procedure.

	LA G (n = 92)	OA G (n = 93)	p
Age (years)	30.7 (8.8–84.1)	39.4 (6.1–87.7)	0.037*
Gender (F/M)	54/38	58/35	0.653†
CRP (mg/dl)	1.91 (0.02–28.8)	3.9 (0.01–30.3)	0.024*
WBC (10^3^/μL)	12.3 (4.3–26.5)	13.0 (4.4–36.4)	0.160*
Alb (g/dL)	4.4 (2.8–5.3)	4.4 (2.5–5.3)	0.154*
Neutro. (%)	80.9 (43.0–95.5)	83.6 (27.6–95.5)	0.674*
Lymph (%)	13.0 (3–46)	10.9 (3–57.8)	0.666*
NLR	6.4 (0.9–31.8)	7.9 (0.5–31.8)	0.725*
DDO	(0–42)	(0–9)	0.056*
Interval appendectomy (yes/no)	7/85	2/91	0.085†

Showing medians and interquartile ranges.

*Tested by Mann-Whitney U-test. †Tested by Fisher's exact test. CRP: C-reactive protein, WBC: white cell count, Alb: Albumin, Neutro: neutrophil, Lymph: lymphocyte. NLR: Neutrophil/Lymphocyte ratio, DDO: day from diagnosis to operation.

**Table 2:** The outcomes according to the procedure.

	LAG (n = 92)	OAG (n = 93)	p
Operating time (min.)	61.5 (28–219)	64 (34–150)	0.670*
Blood loss (g)	1 (1–300)	1 (1–848)	0.038*
LHS (days)	5 (2–24)	7 (3–36)	<0.001*
SOI (days)	1 (0–11)	1 (1–14)	0.016*
SSI (%)	0	4.3	0.044†
Gangrenous (%)	32.6	54.8	0.003†

Showing medians and interquartile ranges.

*Tested by Mann-Whitney U-test. †Tested by Fisher's exact test.

LHS: length of hospital stay, SOI: started an oral intake, SSI: surgical site infection, Clavien-Dindo classification IIIa.

In multivariate analysis, blood loss and LHS were significantly lower in LA G than in OA G (P = 0.038 and 0.023, Table 3).

**Table 3:** Multivariate Analysis clinical and operative factors according to the procedure.

	LAG (n = 92)	OAG (n = 93)	Odd ratio	95% C.I.	p
Blood loss (g)	1 (1–300)	1 (1–848)	2.29	1.05–4.99	0.038^#^
LHS (days)	5 (2–24)	7 (3–36)	2.03	1.11–3.74	0.023^#^

#Tested by logistic regression model analyses.

## 4 Discussion

The first report of laparoscopic appendectomy performed in Taiwan was published in 1999 by Yao et al. [9]. Since 2000, laparoscopic appendectomies have become popular worldwide because they were demonstrated in comparative cases to be well tolerated [10, 11, 12, 13]. Laparoscopic surgical procedure has advantages in several surgical areas of daily practice as a minimally invasive technique, and several reports published in the last decade described the superiority of LA against conventional open approach cosmetic outcomes and low cost also is another factor where LA supersedes open approach, and are an important issues in OA [6, 11]. In addition, less postoperative pain, early recovery, and improved cosmetic appearance are accepted as the main advantages of LA [10]. The aim of this study was to retrospectively evaluate the outcome, such as preoperative characteristics, surgical factors, and postoperative hospital stay, of LA in the treatment of acute appendicitis in comparison with the open approach. At our medical center LA was started in 2010, and now a days, the vast majority of appendicitis cases, are operated laparoscopically.

Longer operative time and massive blood loss during LA are another issue in the comparison of LA and OA. Generally, those two factors are dependent on surgeon's experience. Though most surgical staffs in general has performed basic and advanced laparoscopic procedures, operating time is long when performed by inexperienced surgeons, and is shortened by accumulating experience [10, 14]. Also blood loss is dependent on surgeon's skill and on the situation of appendicitis. In our study, amount of blood loss was significantly lower in LA procedure.

Currently in Japan, shorting postoperative hospital stay is one of the most important factors for economic management of a medical institution, and in order to shorten postoperative hospital stay, surgeons are required to reduce the risk of postoperative complications as much as to their ability. Among the postoperative complications, SSI is the most problematic in terms of extending hospitalization for patients with appendectomy. Consistent with other studies, SSI occurred more often in the OA group [15, 16, 17]. In the previous reports that featured LA as a treatment for acute appendicitis, SSI was not as prominent, and the hospital stay has been clearly shortened [14, 18]. The main reasons for the major number of SSI in the OA group might be due to the direct physical touch to the wound, and the fact that in all of the cases that underwent LA the specimen were removed using a plastic bag. Similar to our results, SSI ratio was zero percent in LA group, and there were no cases of re-admission due to postoperative ileus and/or abscess formation. Furthermore, SOI tended to be short in LA group, and it was suggested that early improvement of postoperative nutritional status would be affecting positively to the decrease of SSI incidence rate. Finally, the low SSI incidence rate was thought to have influenced shortening of post operative hospital stay.

Focusing at the result of univariate analysis, it should be noted that the OA group had higher preoperative CRP level and pathological gangrenous cases than in the LA group. This result suggested selection bias by the surgeons. Before 2014, we did not have enough experience of LA; therefore we avoided LA procedure in patients with those types of complicated acute appendicitis. Unfortunately, those two factors were not evident on multivariate analysis. We recommend that if the patients have those two factors before operation, surgeons should be careful in considering LA or OA as the treatment of choice.

Recent year, several medical centers selected nonsurgical treatment of appendiceal phlegmonous or abscess, using antibiotics treatment, or interval appendectomy. Darwazeh G et al revealed in their systemic review report that interval appendectomy and repeated nonsurgical management in case of recurrence are associated with similar morbidity in emergency appendectomy, but interval appendectomy tended to be high in cost and tend to prolong hospital stay [19, 20]. From our results, 7 cases had interval operation in LA group, and two cases in OA group, but there were no significant differences between the both groups. On the other hand, interval appendectomy cases were slightly high in numbers in the LA group. We suggested and suspected that interval LA cases will be increasing in the next decade with improving laparoscopic technique. Even so, there will be possibility that cost can be reduced and hospital stay can be shortened. For complicated appendicitis (perforation, localized pus or four quadrant pus) it may also be necessary to choose interval surgery. Based on our result, we will prepare diagnostic criteria for complicated appendicitis, and make protocol of interval surgery to prove the effectiveness of interval LA. In conclusion, LA constitutes a safe and feasible procedure for the treatment of acute appendicitis. Furthermore, we have demonstrated that LA is associated with significantly lower risk compared to OA.

## Declarations

### Author contribution statement

Mitsugi Shimoda: Conceived and designed the experiments; Performed the experiments; Analyzed and interpreted the data; Wrote the paper.

Tsunehiko Maruyama: Performed the experiments; Analyzed and interpreted the data.

Kiyotaka Nishida, Kazuomi Suzuki, Tomoya Tago, Jiro Shimazaki: Contributed reagents, materials, analysis tools or data.

Shuji Suzuki: Conceived and designed the experiments; Performed the experiments; Analyzed and interpreted the data.

### Funding statement

This research did not receive any specific grant from funding agencies in the public, commercial, or not-for-profit sectors.

### Competing interest statement

The authors declare no conflict of interest.

### Additional information

No additional information is available for this paper.
